# Psychological, Pain, and Disability Factors Influencing the Perception of Improvement/Recovery from Physiotherapy in Patients with Chronic Musculoskeletal Pain: A Cross-Sectional Study

**DOI:** 10.3390/healthcare12010012

**Published:** 2023-12-20

**Authors:** Roy La Touche, Joaquín Pardo-Montero, Mónica Grande-Alonso, Alba Paris-Alemany, Diego Miñambres-Martín, Encarnación Nouvilas-Pallejà

**Affiliations:** 1 Departamento de Fisioterapia, Centro Superior de Estudios Universitarios (CSEU) La Salle, Universidad Autonoma de Madrid, 28023 Madrid, Spain; roylatouche@yahoo.es (R.L.T.);; 2 Motion in Brains Research Group, Institute of Neuroscience and Sciences of the Movement (INCIMOV), Centro Superior de Estudios Universitarios (CSEU) La Salle, Universidad Autonoma de Madrid, 28023 Madrid, Spain; 3 Instituto de Dolor Craneofacial y Neuromusculoesquelético (INDCRAN), 28008 Madrid, Spain; 4 Departamento de Cirugía, Ciencias Médicas y Sociales, Facultad de Medicina, Universidad de Alcalá, 28871 Alcalá de Henares, Spain; monica.grande@uah.es; 5 Grupo de Investigación Clínico-Docente Sobre Ciencias de la Rehabilitación (INDOCLIN), Centro Superior de Estudios Universitarios La Salle, 28023 Madrid, Spain; 6 Departamento de Radiología, Rehabilitación y Fisioterapia, Facultad de Enfermería, Fisioterapia y Podología, Universidad Complutense de Madrid, 28040 Madrid, Spain; 7 Premium Madrid Global Health Care, 28016 Madrid, Spain; 8 Faculty of Sport Sciences, Universidad Europea de Madrid, 28670 Villaviciosa de Odón, Spain; 9 Department of Social and Organizational Psychology, National University of Distance Education, 28040 Madrid, Spain

**Keywords:** subjective perception of improvement, chronic musculoskeletal pain, therapeutic alliance, self-efficacy, psychological factors, disability

## Abstract

Objectives: The aim of this study was to identify the possible relationships between psychological, pain, and disability variables with respect to the perception of change/recovery from physiotherapy in patients with chronic musculoskeletal pain (CMP). Methods: A cross-sectional observational study was performed with 150 patients. All patients completed a series of self-administered questionnaires and a series of self-reports to quantify the perception of change with respect to the physiotherapy they underwent, the level of disability and pain intensity, the level of fear of movement, the level of catastrophism, the degree of self-efficacy, the level of therapeutic alliance and their adherence to the physiotherapy. Results: The strongest correlations were between the subjective perception of change and the number of sessions, treatment beliefs, self-efficacy, pain intensity, collaboration, and bonding. The linear regression model showed that the number of sessions, treatment beliefs, self-efficacy, compliance, pain intensity, and bonding were predictors of subjective perception of improvement, with 50% of the variance. Conclusions: Treatment beliefs, therapeutic alliance, degree of self-efficacy, and pain intensity have been shown to be predictors of a subjective perception of improvement in patients with CMP. In turn, multimodal treatments had the greatest positive impact on the subjective perception of improvement.

## 1. Introduction

There are various ways to measure the results of treatment; in addition to the objective improvement in pain and in the level of disability, it is important that patients perceive this improvement [1]. Patient-centered care requires that clinicians qualitatively assess the effectiveness of the interventions, asking patients whether their condition has improved. Although this oral self-report is of considerable usefulness, quantifying the perception of improvement with patient-reported outcome measures appears to be necessary [2].

In general terms, measures of patient improvement provide a record of the subjective perception of perceived change over patient’s condition or health status over time [3]. The instruments that assess the patient’s perception of improvement are known specifically as Global Rating of Change (GRC) scales and can have differing numbers of classification levels (between 11 and 15 levels; there are also ones with seven levels) and can vary in their naming. Some of the most commonly mentioned are the Global Rating of Perceived Change (GRPC), the Patients’ Global Impression of Change, and the Global Improvement Scale [4].

GRCs have been employed to measure the subjective perspective of improvement with physiotherapy applied to various chronic musculoskeletal disorders. This assessment provides valuable insights into the effectiveness of the physiotherapy interventions from the patient’s points of view, and helps physiotherapists understand how the patient perceives the impact of treatment [2,5,6]. A number of authors have suggested that these types of scales are valuable for assessing the results of physiotherapy because they require little time to administer, which makes them ideal instruments for the clinical setting [7].

Various studies have reported a number of limitations that can be present in GRC-related results; an important one to consider is the possible influence of “memory bias” or “recall bias” [4,5,8]. Studies have also detected reliability problems when using GRCs in long-term measurements [9]. Studies have suggested that the results of GRCs reflect the perception of the current state and not the perception of change based on the initial state [3]. Studies have also observed that this measure has a poor correlation with functional variables [10,11].

Numerous studies that have analyzed the correlations between GRCs and various clinical variables have found correlations between psychological variables and mood [12,13], depressive signs, kinesiophobia, and pain catastrophism [12], as well as with maladaptive pain-related beliefs [14], disability [2,14,15], and patient satisfaction [16,17].

With regard to pain and GRCs, the data are conflicting; there is evidence indicating weak [7] and moderate negative correlations [15,17]. Another study, however, concluded that the improvements in pain appear not to have such an influence on the GRCs [13].

An especially relevant variable is therapeutic alliance which refers to a trust-based and collaborative relationship between a healthcare provider and a patient, which is fundamental for the effective delivery of healthcare and favorable treatment outcomes [18]. Higher values in therapeutic alliance have been observed to be weakly associated with self-efficacy, improvements in pain, fear of movement, and general improvements represented in the GRC [19]. The model used in that study also included adherence and treatment compliance; however, these variables present no association [19]. It is important to keep investigating these variables (adherence, compliance) regarding GRCs, given that low compliance is known to be associated with poorer results from physiotherapy [20]. Another variable that can be of interest in the research on the perceived improvements from physiotherapy is the number of sessions. Moreover, identifying the type of physiotherapy that can influence the patient-perceived improvements could be relevant at the clinical level. There are a number of studies that have made these comparisons with various physical therapies applied to patients with chronic musculoskeletal disorders [21,22,23].

The study of perceived improvements with the GRCs and their relationship to other variables is highly extensive, as has been shown above; however, one of the weaknesses of the previously mentioned studies, in our opinion, is that they attempt to identify this relationship with few clinical variables. We therefore considered that including more variables in the study model than have already been investigated could more extensively explain the perception of change/recovery through physiotherapy. A number of authors have suggested that the variables for measuring the results that identify or relate the patient-perceived improvements should be broadened to include domains that reflect overall health, physical health, and the cognitive–emotional and social spheres [24].

Based on the above, this study’s main objective was to identify what factors influence the perception of change/recovery through physiotherapy in patients with chronic musculoskeletal pain (CMP). The second objective was to compare different types of physiotherapy interventions and the perception of change/recovery.

## 2. Materials and Methods

### 2.1. Study Design

An observational cross-sectional study was conducted on patients with CMP. The study was conducted according to the guidelines and recommendations of the Strengthening the Reporting of Observational Studies in Epidemiology (STROBE) declaration [25]. The study also followed the principles of the Declaration of Helsinki [26] and was approved by the ethics committee of the La Salle Superior Center of University Studies (CSEULS-PI-038/2021).

### 2.2. Participants

A total of 180 patients were screened and 150 patients were recruited using a consecutive, nonprobabilistic sampling method from 2 physiotherapy clinics located in Madrid, Spain. Of the 30 patients who were not included, 13 did not want to participate, and 17 did not meet the inclusion criteria. All participants were evaluated by physiotherapists with more than 6 years of experience in managing musculoskeletal disorders, who were not responsible for the treatment of the patient. The screening was performed by the physical therapists in charge of the clinics. The patients were classified with having CMP, which was defined as “persistent or recurring pain that arises as part of a pathological process that directly affects the bones, joints, muscles or related soft tissues” [27].

The patients were selected if they met the following criteria: (1) presence of pain lasting more than 6 months; (2) pain intensity ≥3 points on the numerical pain scale; (3) older than 18 years of age; (4) presence of primary CMP classified as primary chronic pain (cannot be directly attributed to a known disease or damage process) or secondary if caused by a disease or process that directly affects the bones, joints, muscles and/or related soft tissues [28]; (5) and good mastery of the Spanish language; and (6) having undergone at least 4 sessions of physiotherapy in the last month.

The exclusion criteria were (1) having passed more than 10 days since last physiotherapy session; (2) cognitive impairment; (3) presence of psychiatric limitations that prevent participation in the study assessments; (4) inability to provide written informed consent; (5) history of musculoskeletal trauma (e.g., fracture); (6) postsurgical musculoskeletal pain during the 6 previous months; (7) musculoskeletal pain of suspected neurological origin (e.g., stroke); (8) and neoplasia (e.g., breast cancer) and/or referred pain (e.g., referred visceral pain).

### 2.3. Procedure

After granting their consent to participate in the study, all participants were provided a set of questionnaires in the clinic before their treatment session, which included a sociodemographic questionnaire (age, sex, educational level, employment status, weight, and height). The participants also had to complete a number of self-reports to quantify the perception of change with regard to the physiotherapy they were undergoing, the level of disability and pain intensity using the Spanish version of the Graded Chronic Pain Scale (GCPS) [29], the level of fear of movement using the Tampa Scale of Kinesiophobia (TSK-11) [30], the level of catastrophism with the chronic pain catastrophism scale [31], the degree of self-efficacy with the Self-Efficacy in Chronic Pain questionnaire [32], the level of therapeutic alliance with the Therapeutic Alliance in Physiotherapy Questionnaire—Patients (CAF-P) [33] and the treatment adherence regarding physiotherapy [34]. The type of treatment and number of sessions received were registered by the assessors consulting the treatment notes of the patients. The assessors also ensured that all the questionnaires were fully completed.

### 2.4. Variables

#### 2.4.1. Main Variable

- Overall perception of improvement

The study employed the Global Rating of Perceived Change (GRPC) [4,35] to assess the patients’ overall perception of improvement. This scale seeks to have the patient self-evaluate their current health condition compared with their previous state. The magnitude of change readings are indicated through an 11-point analog scale, with a middle point (0, unchanged) and 2 ends: the left (−5, much worse) and the right (+5, completely recovered). In this study, the patients were asked to assess the magnitude of change with the following general premise: “Please assess the improvement you experienced since you started the treatment”. The GRPC presents excellent reliability [4,5] and sensitivity to change [4]. The results of the GRPC are categorized into levels to describe the improvements and cross them with other variables: 0 indicates no change; 1 and 2 indicate slight recover; 3 and 4 indicate moderate recovery; 5 indicates complete recovery; −1 and −2 indicate slightly worse; −3 and −4 indicate moderately worse; and −5 indicates much worse. The results of this scale were analyzed both quantitatively and categorically.

#### 2.4.2. Secondary Variables

- Disability

Disability and pain intensity were assessed with the Spanish version of the GCPS, which has been used to measure the degree of interference by chronic pain in daily life activities. The scale consists of 8 items with response options in an 11-point Likert format with a total range of 0–70 points. The scale has 2 subscales, one that measures pain intensity and the other disability. Additionally, the scale rates the disability in moderate and severe levels. This version of the scale has shown good internal consistency (Cronbach’s α of 0.87) [29].

- Therapeutic alliance in physiotherapy

Therapeutic alliance with the physiotherapy was assessed with the CAF-P [33], a self-reporting instrument that assesses the patient’s perception of the therapeutic alliance with the physiotherapist. This questionnaire consists of 19 items, 2 subscales (bonding and collaboration) and a Likert scale with 5 options. The CAF-P has recently been validated and presents excellent internal consistency (Cronbach’s α of 0.91).

- Treatment adherence

Treatment adherence to the physiotherapy was assessed with the Treatment Adherence Scale (AdT). In its validation process, the scale showed excellent internal consistency (Cronbach’s α of 0.897) [34].

- Self-efficacy when dealing with chronic pain

The level of self-efficacy was evaluated using the Spanish version of the Chronic Pain Self-efficacy Scale questionnaire, which has acceptable psychometric properties to assess the perceived self-efficacy and capacity for coping with the consequences of chronic pain (Cronbach’s α of 0.91) [32]. The questionnaire consists of 19 items divided into three dimensions: self-efficacy in controlling symptoms, self-efficacy in handling the pain and self-efficacy in physical activities. The final score ranges from 0 to 190 and is obtained by adding the scores of the three dimensions, with higher scores indicating greater self-efficacy [32].

- Fear of movement

Fear of movement was measured using the Spanish version of the TSK-11, which presents adequate psychometric properties and good internal consistency (Cronbach’s α of 0.81) [30]. The scale consists of 2 subscales, one related to the fear of physical activity and the other related to the fear of injury. Each of the 11 items is scored from 1 to 4 (1 = “completely disagree”, 2 = “disagree”, 3 = “agree”, 4 = “completely agree”). The total scores therefore range from 11 to 44, with higher scores indicating greater fear of movement.

- Pain catastrophism

To measure the degree of catastrophism when faced with painful experiences, the study employed the Spanish version of the Pain Catastrophism Scale, which is a reliable measuring tool (interclass correlation, 0.84) and shows adequate internal consistency (Cronbach’s α of 0.79) [31]. The scale contains 13 items, which are divided into 3 domains: rumination (consistent preoccupation and inability to inhibit pain-related thoughts, 4 items), magnification (exaggeration of the unpleasantness of the pain, 3 items), and hopelessness (loss of hope in achieving anything or that some physical and/or psychological aspect harmful to the health will disappear, 6 items) [31]. A higher score in the scale is considered a higher grade of catastrophism when faced with pain.

### 2.5. Sample Size Calculation

The sample size was calculated with G*Power 3.1.7 (G*Power of the University of Düsseldorf, Germany) [36]. A preliminary pilot study involving 85 participants with chronic musculoskeletal pain was conducted to perform the sample size calculation for multiple regression analysis. This analysis considered the use of 15 predictor variables, which were established from the 7 measurement instruments utilized to address the study objectives, along with each of their sub-scales. The results from this pilot study, employing multiple regression, yielded a squared multiple correlation coefficient (*R*^2^) of 0.2, which led us to estimate an effect size (f^2^) of 0.25. Additionally, the sample size calculation employed an alpha error of 0.05 and a statistical power of 95% (error 1-B). Based on the analysis, the total sample size was determined to be 125 patients; considering the 15 predictor variables, we increased the final sample size to 150 patients (10 patients per variable introduced into the model). This calculation exceeds the calculation recommended by a number of authors who suggest including at least 2 participants per variable for regression analysis [37].

### 2.6. Statistical Analysis

All analyses were performed with the statistical program SPSS version 27.0 (SPSS Inc., Chicago, IL, USA), with a 95% confidence level, considering statistically significant those *p* values < 0.05.

The descriptive statistics are presented as mean, standard deviation and range for the continuous variables; the categorical variables are presented as frequencies and percentages.

The relationship between GRPC and the other predictors was analyzed using Pearson’s correlation coefficients. A Pearson’s correlation coefficient greater then 0.60 indicated a strong correlation, a coefficient between 0.30 and 0.60 indicated a moderate correlation, and a coefficient less than 0.30 indicated a low or very low correlation [38].

A multiple linear regression analysis was employed to estimate the strength of the association between the overall perception of improvement with the GRPC (criterion variable) and the previously mentioned psychological self-reports (predictor variables). The variance inflation factor (VIF) was calculated to determine whether any of the models presented multicollinearity. The value of VIF close to 1 means no multicollinearity (predictor variable independent), 1 < VIF < 5 indicates moderate multicollinearity (predictor variables are moderately correlated to each other), VIF > 10 indicates presence of multicollinearity (predictor variables are dependent and highly associated to each other) [39]. The strength of the association was examined using regression coefficients (β), *p*-values and adjusted R^2^. We report the β coefficients standardized for each prediction variable included in the final models to allow for the direct comparison between the prediction variables in the regression model and the criterion variable.

In assessing the viability of our multiple linear regression model, we incorporated several diagnostic analyses.

- Homogeneity was evaluated visually using a residual versus fitted values plot. The spread of residuals was random without discernible patterns or funnel shapes, indicating homogeneity of variance.
- The assumption of the Independence of observations was tested using the Durbin–Watson statistic, which helps detect autocorrelation in the residuals of regression analysis. A Durbin–Watson statistic value close to 2.0 suggested no evidence of autocorrelation in our model.
- To assess the Normality of the distribution, Q-Q plots of the residuals were examined. The residuals approximated a straight line, which is consistent with a normal distribution.
- Linearity was verified through an analysis of scatter plots comparing observed versus predicted values, as well as through the examination of normal P-P plots of standardized residuals. These plots demonstrated a linear relationship, affirming the linearity assumption of our model.

An analysis was performed with contingency tables using the GRPC as the categorical variable and the treatment type variable with the chi-squared test.

Finally, percentages were presented based on the overall perception of improvement in relation to the treatments received by the patients. Contingency table analysis was conducted using the chi-squared test to compare the observed frequencies. The perception of improvement was categorized from no change to complete recovery.

## 3. Results

The final sample consisted of 150 patients with CMP, a total of 115 women and 35 men with a mean age of 52.69 ± 14.87 years. It is important to note that there were no dropouts. The statistics of the clinical and demographic variables are presented in Table 1.

The results from the diagnostic analyses conducted to assess the validity of our multiple linear regression model indicate that the model is appropriate. Homoscedasticity has been confirmed, as evidenced by the random scatter of residuals without any discernible patterns. The Durbin–Watson statistic yielded a value of 2.13, suggesting an absence of autocorrelation within the model. Additionally, the normal P-P plot of standardized residuals suggests a linear relationship. Regarding the normality assessment, the Q-Q plot revealed that five variables did not adhere to a normal distribution. Specifically, the three subscales pertaining to pain catastrophizing, disability, and the number of sessions.

### 3.1. Correlation Analysis

Table 2 shows the descriptive statistics of the clinical self-reports and correlation analysis. The strongest correlations with the GRPC were with the number of sessions (r = 0.45; *p* < 0.01), treatment beliefs (r = 0.48; *p* < 0.01), self-efficacy for managing pain (r = 0.39; *p* < 0.01), pain intensity (r = 0.33; *p* < 0.01), collaboration (r = 0.37; *p* < 0.01) and bonding (r = 0.42; *p* < 0.01).

### 3.2. Regression Analysis

Table 3 presents the multiple linear regression model for the GRPC criterion variable. In the model, we found that the number of sessions, treatment beliefs (AdT-Physio subscale), self-efficacy for managing pain, compliance (AdT-Physio subscale), pain intensity (GRPC subscale) and bonding (CAF-P subscale) were predictors of GRPC, which explained 50% of the variance. These variables account for a portion of the reasons (up to a 50%) why patients perceived change. Nine variables were excluded from the model. The VIF analysis indicated that the obtained model presented very low multicollinearity as evidenced by a value close to 1. This suggests that the predictor variables are not strongly associated, signifying that each of the predictor variable has an independent effect on the criterion variable.

### 3.3. Contingency Table Analysis

**Figure 1:**
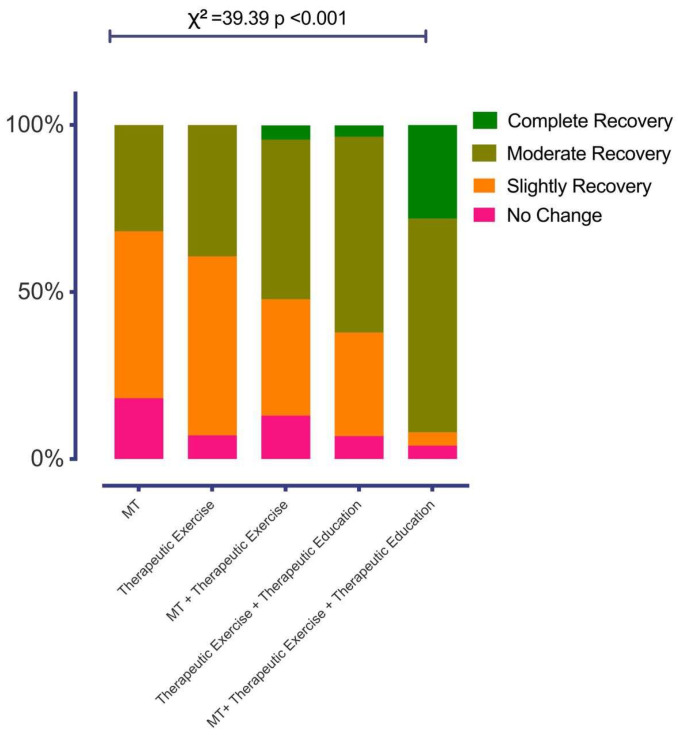
The figure represents the percentages according to the overall perception of improvement in relation to the treatments received by the patients.

In the analysis of percentages between the treatment type and the GRPC in a categorical result, there were statistically significant differences (χ^2^ = 39.39; *p* < 0.001). Figure 1 shows that for the overall perception categories of “completely recovered” and “moderately recovered” the percentages are higher in the treatments of “exercise + education” and “manipulative therapy + exercise + education” compared with “manipulative therapy”, “exercise” and “manipulative therapy + exercise”.

## 4. Discussion

The aim of this study was to identify the clinical variables (psychological, disability, pain, and treatment) related to the patient’s perception of improvement as measured with the GRPC. The results of the study indicate that, as a whole, the treatment beliefs, number of sessions, self-efficacy in managing pain, the bonding as part of therapeutic alliance in physiotherapy, compliance with physiotherapy, and pain intensity explain the 50% variance of the GRPC. When confirming the second objective, we observed that the multimodal or combined physiotherapy (exercise, education, and manipulative therapy) presented higher percentages of recovery perception specifically in the options of “moderately recovered” and “completely recovered”.

The model accepted in the regression analysis showed the inclusion of a significant number of variables (6 of them) and an explained variance of 50%, which could be due to the possible multidimensionality of the GRPC and, as suggested by a number of authors, the perception of improvement should be assessed beyond the measures of pain and disability [24]. The results lead to the consideration that the construct of the patient’s perception of improvement/recovery includes other variables that could be important but have not been identified in this study. Additionally, it has been shown that self-reported psychological measures in patients with pain considerably overlap [40].

A number of the variables identified in the model such as pain intensity, therapeutic alliance (in our case, a subscale of bonding) and self-efficacy in managing pain coincide partially with the findings of previous studies [7,15,17,19] and are in contrast to certain variables such as levels of disability [2,14,15], pain catastrophism and fear of movement [12]. These were identified in previous studies but were not included in our study’s model, perhaps because self-efficacy, catastrophism, and kinesiophobia might be part of the same underlying psychological construct that we can term “pain-related distress” [40].

It is also important to note the time at which the predictors of patient improvement (GRPC) are measured, given that Meisingset et al. (2018) [41] mentions that, at baseline, the psychological factors (kinesiophobia, catastrophism and self-efficacy) are not predictors, although they are when evaluating the change in the levels of these variables due to the treatment [41].

The treatment beliefs and compliance (adherence subscales) and the number of sessions are variables that have been identified in the model and, to our knowledge, have not been identified in previous studies. In terms of the treatment beliefs, it is important to note that the items of this subscale are written as possible beliefs or positive expectations regarding the physiotherapy. We assume that this variable has a more significant influence in the model due to affective-motivational aspects related to the physiotherapy performed at the time, which has positively influenced the perception of recovery. A number of representative items in this subscale are proposed in these terms of satisfaction/motivation: “the physiotherapy I’m performing is well-structured”; “following the guidelines indicated by the physiotherapist improves my health”; and “the motivation offered me by the physiotherapist to perform the exercises is appropriate” [34]. In a cross-sectional study, it is logical that the positive beliefs regarding treatment are related to the perception of improvement.

The patients can assess various aspects in their judgment of their improvement. For example, in a study by Evans et al. (2014) [42] with a sample of patients with neck conditions, some of the patients focused on their judgment of typical aspects related to the intervention such as the information regarding the treatment and the performing of specific exercises; other focused on being supervised, while others focused on the perceived improvement in biomechanical variables such as strength and range of motion [42]. Therefore, as stated by Beaton et al., “the perception of improvement is highly contextualized in the individual’s experience”. This interpretation can be affected by the state of the disorder or disease (resolution), the potential adjustments to life that favor the changes in the disorder (readjustment) and in adapting to living with the disorder (redefinition) [43]. We assume that cognitive variables such as treatment beliefs, self-efficacy, treatment adherence and other affective types of variables such as bonding can function as mediators of the resolution, the readjustment and the redefinition; however, this suggestion is clearly theoretical and should be the subject of future research.

The patient’s judgment of their state of health is influenced by their values and preferences as components of their own experience. Further research is needed on how to assess and improve their state of health according to the patient’s individual characteristics [44].

It is of interest to analyze the role that bonding could play within the therapeutic alliance and as a possible mediator of the perception of improvement/recovery. As mentioned previously, bonding is a variable encompassed by an affective dimension and “reflects the emotions and attitudes that the patient and the therapist have between them and enable the therapeutic work through a sense of collaboration and confidence” [45]. Previous reviews have reported that a greater therapeutic alliance is associated with greater adherence and have indicated that motivation and support for autonomy appear to be facilitators of the therapeutic alliance [46]. A strong therapeutic alliance can promote pain reduction in patients with CMP [18], a corroborated fact that might be extended in turn to the perception of improvement/recovery with the treatment. Ferreira et al. verified that the therapeutic alliance was a moderator of the overall perception of the effect of physiotherapy in patients with chronic low back pain [47].

Lastly, it is worth mentioning the positive correlation observed between the number of physiotherapy sessions undergone and the GRPC. As an important consideration of this aspect, we need to mention that it could be due to the type of study design and the inclusion criteria established at the start of the study, the sample and number of sessions attended varied significantly. The minimum number of sessions that the patients should have undergone was 4; however, in many cases this number was exceeded. Moreover, the patients who comprised the total sample had CMP, and certain aspects of the physiotherapy require time to achieve specific adaptations. It is therefore highly possible that this is one of the reasons why the patients who performed more sessions had better GRPC. For example, therapeutic alliance requires time to establish.

As described in previous studies [21,22,23] and confirmed in this study, multimodal physiotherapy generates higher rates of perceptions of improvement. Specifically the exercise treatment, education and manipulative therapy showed higher percentages of “completely recovered” and “moderately recovered”, a result that is very similar to those of the study by López-de-Uralde-Villanueva [21]. In this study, the manual therapy interventions included articular mobilizations and conservative soft tissue techniques; the therapeutic exercise included local stabilization and mobilization exercises and well as general strength exercises; therapeutic education was focused in pain neurophysiology education and pain coping strategies.

### Limitations

This study has limitations that should be considered when interpreting the results. The first is that this study has a cross-sectional observational design and was therefore not able to control many of the treatment-related variables such as the total number of sessions and the type of treatment applied. To solve this limitation, future studies should employ longitudinal designs of a cohort type or with randomized controlled clinical trials. Another limitation is that mood-related outcome measures were not included; these measures are highly related to the perception of improvement/recovery [18].

The sample consisted mostly of individuals with university degrees, which does not represent the distribution of the Spanish population. Future studies could also concurrently assess the patient’s perception of improvement using other complementary methods such as with semi-structured interviews as employed by Evan et al. 2014 [42].

Lastly, we should also consider as a limitation not having controlled the professional characteristics of the physiotherapists who performed the treatments (years of study, type of specialized training, and experience).

## 5. Conclusions

In conclusion, the results of the study indicate that the combination of treatment beliefs, number of sessions, self-efficacy in managing pain, bonding as part of therapeutic alliance in physiotherapy, compliance with physiotherapy, and pain intensity explain the 50% variance of the GRPC. We also observed that multimodal physiotherapy based on therapeutic education, therapeutic exercise and manipulative therapy had a higher subjective perception of improvement in the patients with CMP.

## Figures and Tables

**Table 1:** Descriptive statistics for demographic outcomes.

Measures	Mean ± SD/Number (%)	Minimum-Maximum
Age	56.9 ± 14.08	22–80
BMI	25.67 ± 4.42	18.36–41.24
Number of sessions	10.33 ± 5.33	4–35
Sex		
- Female	115 (76.7)	
- Male	35 (23.3)	
Educational level		
- Obligatory education	10 (6.7)	
- High School/PT	45 (30)	
- University education	95 (63.3)	
Employment status		
- Active worker	72 (48)	
- Not active worker	12 (8)	
- Sick leave—short-term	33 (22)	
- Retired—due to age	33 (22)	
Types of treatment		
- Manual therapy	22 (14.7)	
- Therapeutic exercise	28 (18.7)	
- Manual therapy + therapeutic exercise	46 (30.7)	
- Therapeutic exercise + therapeutic education	29 (19.3)	
- Manual therapy + therapeutic exercise+ therapeutic education	25 (16.7)	

BMI: Body Mass Index; PT: professional training.

**Table 2:** Correlation analysis.

Measures	Mean ± SD	1	2	3	4	5	6	7	8	9	10	11	12	13	14	15
GRPC	2.45 ± 1.33	1														
2. Number of sessions	10.33 ± 5.33	0.45 ***														
3. GCPS_Intensity of pain	21.64 ± 6.96	−0.33 ***	−0.171*													
4. GCPS_Disability	16.21 ± 9.91	−0.14	−0.04	0.42 ***												
5. CAF-P_collaboration	34.76 ± 9.17	0.37 ***	0.15	−0.08	−0.11											
6. CAF-P_bonding	13.14 ± 3.85	0.42 ***	0.15	−0.09	−0.03	0.58 ***										
7. AdT-Physio_compliance	26.64 ± 4.48	0.04	0.06	0.05	0.11	0.32 ***	0.27 ***									
8. AdT-Physio Beliefs about treatment	21 ± 4.8	0.48 ***	0.22 **	−0.11	−0.01	0.41 ***	0.39 ***	0.44 ***								
9. CPSS_controlling symptoms	57.31 ± 13.83	0.14	0.07	−0.04	−0.22 **	0.10	0.19 *	0.19 *	0.03							
10. CPSS_physical activities	49.19 ± 12.61	0.12	0.15	−0.14	−0.34 ***	0.01	0.12	0.04	−0.01	0.70 ***						
11. CPSS_handling the pain	32.77 ± 12.64	0.39 ***	0.21 **	−0.35 ***	−0.37 ***	0.19 *	0.21 *	0.03	0.12	0.50 ***	0.55 ***					
12. TSK_fear of injury	10.88 ± 3.14	−0.17 *	−0.09	0.13	0.38 ***	−0.07	−0.09	0.06	−0.07	−0.20 *	−0.30 ***	−0.12				
13. TSK_fear of physical activity	17.32 ± 5.44	−0.15	−0.11	0.18 *	0.51 ***	0.09	−0.01	0.17 *	−0.02	−0.25 **	−0.42 ***	−0.19 *	0.70 ***			
14. PCS_Rumination	5.35 ± 4.21	−0.24 *	−0.14	0.36 ***	0.47 ***	−0.05	−0.15	−0.01	−0.02	−0.30 ***	−0.25 **	−0.37 ***	0.17 *	0.30 ***		
15. PCS_magnification	2.97 ± 2.49	−0.23 **	−0.12	0.25 **	0.42 ***	−0.14	−0.18 *	−0.17 *	−0.09	−0.41 ***	−0.24 **	−0.28 ***	0.29 ***	0.31 ***	0.79 ***	
16. PCS_hopelessness	5.83 ± 5.21	−0.23 **	−0.09	0.30 ***	0.50 ***	−0.11	−0.19 *	−0.17 *	−0.10	−0.37 ***	−0.28 ***	−0.29 ***	0.34 ***	0.33 ***	0.72 ***	0.78 ***

* *p* < 0.05; ** *p* < 0.01; *** *p* < 0.001. GRPC: Global Rating of Perceived Change; GCPS: Graded Chronic Pain Scale; TSK: Tampa Scale of kinesiophobia; CPSS: Chronic Pain Self-efficacy Scale; PCS: Pain Catastrophizing Scale; CAF-P: Therapeutic alliance with the physiotherapy; AdT-Physio: Treatment adherence Scale.

**Table 3:** Regression model for GRPC in patients with CMP.

Criteria Variable: GRPC				
General Model				
R^2^ = 0.52; Adjusted R^2^ = 0.50; F = 26.32; *p* < 0.01				
Predictor Variables	Regression Coefficient (B)	Standardized Coefficient (B)	*p* Value	VIF
AdT-Physio Beliefs about treatment	0.13	−0.48	<0.001	1
Number of sessions	0.09	0.35	<0.001	1.05
CPSS_handling the pain	0.03	0.28	<0.001	1.08
CAF-P_bonding	0.07	0.20	0.003	1.21
AdT-Physio_compliance	−0.06	−0.21	0.001	1.26
GCPS_Intensity of pain	−0.03	−0.18	0.006	1.19
**Excluded variables**				
CAF-P_collaboration	-	0.11	0.14	1.66
TSK_fear of physical activity	-	−0.02	0.67	1.11
TSK_fear of injury	-	−0.03	0.53	1.05
CPSS_controlling symptoms	-	−0.01	0.9	1.44
CPSS_physical activities	-	−0.09	0.2	1.57
PCS_Rumination	-	−0.01	0.82	1.32
PCS_magnification	-	−0.05	0.41	1.19
PCS_hopelessness	-	0.04	0.52	1.31
GCPS_Disability	-	0.11	0.09	1.25

CMP: Chronic musculoskeletal Pain; VIF: Variance Inflation Factor; GRPC: Global Rating of Perceived Change; GCPS: Graded Chronic Pain Scale; TSK: Tampa Scale of kinesiophobia; CPSS: Chronic Pain Self-efficacy Scale; PCS: Pain Catastrophizing Scale; CAF-P: Therapeutic alliance with the physiotherapy; AdT-Physio: Treatment adherence Scale.

## Data Availability

Data supporting reported results will be available under request to the corresponding author.
